# Insulin-like growth factor-I and prostate cancer: a meta-analysis

**DOI:** 10.1054/bjoc.2001.1961

**Published:** 2001-09

**Authors:** R Shi, H J Berkel, H Yu

**Affiliations:** Section of Cancer Prevention and Control, Feist-Weiller Cancer Center, LSUHSC-Shreveport; Department of Medicine, Louisiana State University Health Sciences Center – Shreveport, 1501 Kings Highway, Shreveport, LA, 71130-3932, USA

**Keywords:** IGF-I, prostate cancer, meta-analysis, case–control studies, odds ratio

## Abstract

Some, but not all, epidemiological found have shown that high circulating levels of insulin-like growth factor-I (IGF-I) are associated with an increased risk of prostate cancer. We performed a meta-analysis on all the studies reported so far to evaluate this association. In our Medline search, 14 case–control studies were identified. A standard protocol abstracted information for each study. Hedges' standardized mean difference (HSMD) and odds ratio (OR) were used to estimate the effect of IGF-I and IGF-binding proteins (IGFBP-3). The combined data showed that circulating levels of IGF-I were significantly higher in prostate cancer patients (HSMD = 0.194). The OR for prostate cancer was 1.47 (95% confidence interval (CI) 1.23–1.77) among men with high IGF-I compared to those with low IGF-I. The OR was 1.26 (95% CI 1.03–1.54) for IGFBP-3. Circulating levels of IGF-I and IGFBP-3 are likely to be higher in prostate cancer patients than in the controls. These findings support the suggestion that high IGF-I and IGFBP-3 are associated with an increased risk of prostate cancer. © 2001 Cancer Research Campaignhttp://www.bjcancer.com


British Journal of Cancer (2001) **85**(7), 991–996
© 2001 Cancer Research Campaign
doi: 10.1054/ bjoc.2001.1961, available online at http://www.idealibrary.com on IDEAL

http://www.bjcancer.com

### Insulin-like growth factor-I and prostate cancer: a meta-analysis

**R Shi[1,2], HJ Berkel[1] and H Yu[1]**

[1]Section of Cancer Prevention and Control, Feist-Weiller Cancer Center, LSUHSC-Shreveport, [2]Department of Medicine, Louisiana State University Health Sciences Center – Shreveport, 1501 Kings Highway, Shreveport, LA 71130-3932, USA

**Summary** Some, but not all, epidemiological found have shown that high circulating levels of insulin-like growth factor-I (IGF-I) are associated with an increased risk of prostate cancer. We performed a meta-analysis on all the studies reported so far to evaluate this association. In our Medline search, 14 case–control studies were identified. A standard protocol abstracted information for each study. Hedges' standardized mean difference (HSMD) and odds ratio (OR) were used to estimate the effect of IGF-I and IGF-binding proteins (IGFBP-3). The combined data showed that circulating levels of IGF-I were significantly higher in prostate cancer patients (HSMD = 0.194). The OR for prostate cancer was 1.47 (95% confidence interval (CI) 1.23–1.77) among men with high IGF-I compared to those with low IGF-I. The OR was 1.26 (95% CI 1.03–1.54) for IGFBP-3. Circulating levels of IGF-I and IGFBP-3 are likely to be higher in prostate cancer patients than in the controls. These findings support the suggestion that high IGF-I and IGFBP-3 are associated with an increased risk of prostate cancer. © 2001 Cancer Research Campaign  http://www.bjcancer.com

**Keywords**: IGF-I, prostate cancer, meta-analysis, case–control studies, odds ratio

Insulin-like growth factors (IGFs) are peptide hormones involved in regulation of cell proliferation, differentiation and apoptosis. Most IGFs in the circulation originate in the liver. Since IGFs are regulated by both endocrine and paracrine mechanisms, the action of IGFs in tissue, however, is determined not only by the circulating levels of IGFs and IGF-binding proteins (IGFBPs), but also by local production (Jones and Clemmons, 1995). There is a large interpersonal variability in the circulating concentrations of IGF-I and IGFBP-3 (a major IGFBP in the circulation) (Juul et al, 1994, 1995).

Several studies have shown that prostate cancer patients have higher serum IGFBP-3 levels than healthy males (Cohen et al, 1993; Ho and Baxter, 1997; Wolk et al, 1998; Signorello et al, 1999; Stattin et al, 2000). Some studies have reported that IGF-I levels were higher in prostate cancer patients than in the control subjects (Cohen, 1993; Ho and Baxter, 1997; Mantzoros et al, 1997; Chan et al, 1998; Wolk et al, 1998; Djavan et al, 1999; Signorello et al, 1999; Stattin et al, 2000; Harman et al 2000), whereas others showed the opposite (Kanety et al, 1993; Finne et al, 2000; Kurek et al, 2000).

To further examine the association of IGF-I with prostate cancer, we performed a systematic meta-analysis of all the studies published so far, to determine whether, after controlling for age, circulating levels of IGF-I are higher in prostate cancer patients than in control subjects. We also assess the strength of the association between circulating IGFBP-3 and prostate cancer.

*Received 19 April 2001*
*Revised 21 May 2001*
*Accepted 22 May 2001*

*Correspondence to:* R Shi

### MATERIALS AND METHODS

### Selection of studies

We searched the OVID MEDLINNE database (from January 1966 through February 2001) for articles that had the following text in the subject heading: 'Insulin-like growth factor-I', 'prostate neoplasm;' and in combination with 'case control studies' or 'prospective studies'. We also searched the PubMed database (from the mid-1960s through February 2001) for literature with the keywords 'insulin-like growth factor-I', 'prostate neoplasm', and in combination with 'case control studies' or 'prospective studies'. These searches were restricted to the studies in which IGF-I concentration was measured. We also performed a manual search of references cited in the published original and review articles. Two investigators independently reviewed all potentially relevant articles. Disagreement or uncertainty between two investigators was resolved by discussion. Inclusion was restricted to case–control studies and prospective cohort studies published in English.

### Data abstraction

Data were independently abstracted in duplicate by 2 investigators using a standard protocol and data collection form. Disagreements were resolved by discussion. Characteristics abstracted from the studies included: the name of first author, location of the study, year of publication, case definition, control definition and selection criteria, method of IGF-I and IGFBP-3 measurements, confounding factors that were controlled for by matching or adjustment, and mean and standard deviation of IGF-I and IGFBP-3 in each group.

### Statistical analysis

The meta-analysis methods developed by Hedges and Olkin (1985) were used in our analysis. The following steps were taken in the analysis specifically. First, weighted mean effect sizes (standardized mean level difference) and their 95% confidence intervals (CIs) were calculated. A mean effect size is statistically significant when the CI does not include zero. Second, the homogeneity of effect sizes among these studies was determined. Third, moderator variable analysis was performed to determine whether effect sizes varied significantly with study characteristics. In this study, the type of control (healthy male or patient with benign prostate hyperplasia) and study score (high or low) are the moderator variables. Since sample size varied among the studies, a correction procedure (Hedges and Olkin, 1994) was applied to the standardized mean difference to yield unbiased estimates of their population values. We pooled effect sizes in our analysis using a fixed-effect model because statistical power is higher for the fixed-effect model than for a random-effect model (Rosenthal, 1994). A random-effect model usually generates a more conservative estimate than a fixed-effect model (DerSimonian and Laird, 1986). The homogeneity of the Hedges' standardized mean difference across studies was evaluated by using Hedges' method (Hedges and Olkin, 1985). The chi-square test (Prentice and Thomas, 1986) was used to test homogeneity among studies when the effect size is taken to be the odds ratio (OR).

The effects size of standardized mean difference was classified as small (< 0.2), moderate (0.2–0.5), and large (> 0.5) (Cohen, 1965). For example, a moderate effect size of 0.5 implies that the score of the average individual in the case group exceeds that of 69% of individuals in the control group (Hedges and Olkin, 1985). We also used the OR as an alternative measure of the effect size to evaluate the association between IGF-I and prostate cancer. To calculate the OR, we converted the estimated effect size ($\delta$) to a correlation coefficient ($\rho$) using a method of Hedges and Olkin (1985; Equation 1). Where the $n^{cs}$ is the total number of cases and $n^{ct}$ is the total number of controls. If an effect size is < 0, then a negative value of the correlation coefficient derived from previous step was used in the next step. Next, the proportion of cases that have a higher (greater than the median) IGF-I level (success rate, $0.5 + \rho/2$ for case with higher IGF-I level and $0.5 – \rho/2$ for control with lower IGF-I level) was derived from the Rosenthal and Rubin's criteria (1982). These processes were repeated for the Hedges standardized mean difference (HSMD) and its 95% CI. The OR and its 95% CI were estimated from equation 2. The general variance-based method was used to estimate the summary measure of OR and its 95% CI (Prentice and Thomas, 1986).

$$\rho^2 \cong \frac{\delta^2}{\delta^2 + \dfrac{n^{cs} + n^{ct} - 2}{\dfrac{1}{n^{cs}} + \dfrac{1}{n^{ct}}}} \qquad (1)$$

$$\mathrm{OR} = \left( \frac{0.5 + \dfrac{\rho}{2}}{0.5 - \dfrac{\rho}{2}} \right)^2 \qquad (2)$$

We also examined the influence of various exclusion criteria on the overall standardized mean difference of IGF-I and IGFBP-3 between the cases and controls. One of the exclusion criteria was the use of patients with benign prostatic hyperplasia as controls; another was small sample size (i.e. total < 100). According to the central limit theorem, the distribution of effect-size estimates will be approximately normal if the sample size in each study is large enough (Daniel, 1999). In our analysis, a number of cases or controls > 50 were considered a reasonably large sample size for normal approximation.

Studies in which the controls consisted entirely of benign prostatic hyperplasia (BPH) patients or in which the sample size was < 50 in either case or control groups were classified as a low-score group. Studies in which the controls consisted entirely of healthy males and in which sample size was > 50 in both case and control groups were categorized as a high-score group. Based on these criteria, seven studies (Cohen et al, 1993; Kanety et al, 1993; Ho and Baxter, 1997; Baffa et al, 2000; Djavan et al, 1999; Finne et al, 2000; Khosravi et al, 2001) were classified as low score and the other 7 as high score.

In our meta-analysis, the OR for IGF-I was reported first and followed by IGFBP-3. The estimation of OR by control type was reported first and followed by study score. For the purpose of reference, the corresponding Hedges' standardized mean difference was also reported where appropriate.

### RESULTS

A total of 14 studies were found as a result of our literature search, 12 of which reported the mean and standard deviation of IGF-I. We obtained the mean and standard deviation of IGF-I and IGFBP-3 directly from the authors of the two studies that did not report these data (Chan et al, 1998; Harman et al, 2000). The characteristics of the studies are presented in Table 1. Of the 14 case–control studies (two of them nested), 5 were conducted in the USA, 6 in Europe, and 3 elsewhere. The total number of study subjects ranged from 19 (Ho and Baxter, 1997) to 665 (Finne et al, 2000); the number of cases ranged from 12–210 and the number of controls ranged from 6–486. IGF-I concentrations were determined by radioimmunoassay (RIA) in 6 of the 14 studies (Table 1), while immunoradiometric assay (IRMA) and enzyme-linked immunoabsorbent assay (ELISA) were used in 4 and 4 studies, respectively. IGFBP-3 was measured using IRMA, RIA, and ELISA in 3, 2, and 2 of the 9 studies, respectively; in the other 2 studies it was measured by either Western blots or immunofluorometric assay (IFMA). As a confounding factor, age was controlled in 8 of the 14 studies.

The mean and standard deviation of IGF-I concentration were obtained from all 14 studies, but of IGFBP-3 in only 9. Table 2 lists the sample size and mean concentration of IGF-I and IGFBP-3 in all the studies analysed. The concentration unit for IGF-I was ng/ml in 13 of the 14 studies and nmol/l in the other study (Kanety et al, 1993). For IGFBP-3, the unit was ng/ml in 8 of the 9 studies and unit/ml in 1 study (Cohen et al, 1993). Table 3 lists the comparison of mean age between the case and control groups. Four studies did not report mean and standard deviation of age, but in 3 of the 4 studies, cases and controls were matched on age (± 1 year) (Cohen et al, 1993; Chan et al, 1998; Stattin et al, 2000). There were no statistically significant differences in mean age between the cases and controls in 6 studies, though one study showed a significantly higher mean age in the cases than in controls (Kanety et al, 1993).

The overall estimations of the HSMD for IGF-I for all the studies combined, high-score group, low-score group, studies in

 © 2001 Cancer Research Campaign

**Table 1** Characteristics of case–control studies for prostate cancer and IGF-I and IGFBP-3

| Author | Year and location | Cases | Controls | Method for IGF- I | Method for IGFBP-3 | Variables matched/controlled for |
|---|---|---|---|---|---|---|
| Cohen et al | 1993, North Carolina, USA | 32 untreated prostate cancer | 16 healthy males | RIA | Western blots | age |
| Kanety et al | 1993, Israel | 14 prostate cancer | 6 healthy males | RIA | | |
| Ho and Baxter | 1997, Australia | 12 inoperable PC | 7 healthy males | RIA | RIA | |
| Mantzoros et al | 1997, Massachusetts, USA | 51 histologically confirmed PC | 52 healthy, elderly males | RIA | | age+/–1 |
| Chan et al* | 1998, Maryland, USA | 152 confirmed cases | 152 healthy males | ELISA | ELISA | age+/–1, smoking, follow-up duration |
| Wolk et al | 1998, Sweden | 210 newly, cytologically and historically confirmed PC | 224 biopsy specimens showed no evidence of cancer | IRMA | IRMA | |
| Djavan et al | 1999, Austria | 71 prostate cancer | 174 benign prostate hyperplasia | IRMA | | |
| Signorello et al | 1999, Sweden | 208 prostate cancer | 70 healthy males | IRMA | IRMA | |
| Baffa et al | 2000, Pennsylvania, USA | 57 prostate cancer | 39 males | ELISA | | age |
| Finne et al | 2000, Finland | 179 prostate cancer | 486 benign controls | ELISA | IFMA | |
| Harman et al | 2000, Maryland, USA | 72 prostate cancer | 127 controls | RIA | RIA | age, lengths of follow-ups |
| Kurek et al | 2000, Germany | 171 prostate cancer | 67 males | RIA | | age |
| Stattin et al | 2000, Sweden | 149 prostate cancer | 298 males | IRMA | IRMA | age+/–1, survey date, residency |
| Khosravi et al | 2001, Canada | 84 prostate cancer | 75 benign prostate hyperplasia | ELISA | ELISA | age, |

All are case–control studies, except studies of Chan et al and Harman et al, which is a nested case-referent study.

© 2001 Cancer Research Campaign

**Table 2** Studies reporting IGF-I and IGFBP-3 levels in prostate cancer patients and their controls

| Author and year | N | Case | | Control | | |
|---|---|---|---|---|---|---|
| | | $\overline{X}$ (ng/ml) | SD (ng/ml) | N | $\overline{X}$(ng/ml) | SD(ng/ml) |
| | | IGF-I | | | | |
| Cohen et al, 1993 | 32 | 151 | 42 | 16 | 138 | 31 |
| Kanety et al, 1993 | 14 | 17.9* | 1.7* | 6 | 24.6* | 3.7* |
| Ho and Baxter, 1997 | 12 | 139 | 86.6 | 7 | 117 | 44.98 |
| Mantzoros et al, 1997 | 51 | 160.3 | 68.2 | 52 | 124.7 | 58.6 |
| Chan et al, 1998 | 151 | 269.4 | 85.7 | 152 | 248.9 | 80.9 |
| Wolk et al, 1998 | 210 | 158.4 | 53.8 | 224 | 147.4 | 47.6 |
| Djavan et al, 1999 | 71 | 176 | 26 | 174 | 136 | 23 |
| Signorello et al, 1999 | 208 | 158.2 | 53.8 | 70 | 151.5 | 53.3 |
| Baffa et al, 2000 | 57 | 124.6 | 58.2 | 39 | 157.5 | 70.8 |
| Finne et al, 2000 | 179 | 183 | 66.90 | 486 | 194 | 54.11 |
| Harman et al, 2000 | 71 | 148.3 | 45.2 | 126 | 145.1 | 50.0 |
| Kurek et al, 2000 | 171 | 158.6 | 66.5 | 67 | 159.1 | 58.4 |
| Stattin et al, 2000 | 149 | 229 | 70.27 | 298 | 214.4 | 57.5 |
| Khosravi et al, 2001 | 84 | 126.6 | 44.82 | 75 | 101.2 | 47.2 |
| | | IGFBP-3 | | | | |
| Cohen et al, 1993 | 36 | 13.1† | 0.8† | 18 | 13.2† | 1.8† |
| Ho and Baxter, 1997 | 12 | 2434 | 270 | 7 | 1909 | 364 |
| Chan et al, 1998 | 152 | 2841.7 | 757.9 | 152 | 2836.3 | 794.9 |
| Wolk et al, 1998 | 210 | 2668 | 1037 | 224 | 2518 | 774 |
| Signorello et al, 1999 | 208 | 2664 | 1041 | 70 | 2556 | 783 |
| Finne et al, 2000 | 179 | 4558 | 1337.9 | 486 | 4526 | 1234.5 |
| Harman et al, 2000 | 71 | 2800 | 720 | 126 | 2800 | 760 |
| Stattin et al, 2000 | 149 | 2611 | 578.1 | 298 | 2498.5 | 539.5 |
| Khosravi et al, 2001 | 84 | 2220 | 559 | 75 | 2070 | 528 |

*nmol/1; †units/ml.

**Table 3** Comparison of mean ages in the case and control groups

| Studies | | Case(years) | | | Control(years) | | | |
|---|---|---|---|---|---|---|---|---|
| | | N | $\overline{X}$ | SD | N | $\overline{X}$ | SD | *P*-value* |
| Cohen et al, 1993 | | 32 | – | – | 16 | – | – | – |
| Kanety et al, 1993 | | 14 | 68.5 | 3.4 | 6 | 56.4 | 5.9 | <0.001 |
| Ho and Baxter, 1997 | | 12 | 74.4 | 6.2 | 7 | 68.6 | 7.1 | 0.081 |
| | ≤ 69 | 20 | | | 21 | | | 0.973† |
| Mantzoros, 1997 (Age group) | 70–74 | 18 | | | 17 | | | |
| | ≥ 75 | 14 | | | 14 | | | |
| Chan et al, 1998 | | 152 | – | – | 152 | – | – | – |
| Wolk et al, 1998 | | 210 | – | – | 224 | – | – | – |
| Djavan et al, 1999 | | 71 | 65.7 | 6 | 174 | 67.7 | 9 | 0.086 |
| Signorello et al, 1999 | | 208 | 69.9 | 6.3 | 70 | 70.9 | 5.5 | 0.237 |
| Baffa et al, 2000 | | 57 | – | – | 39 | – | – | – |
| Finne et al, 2000 | | 179 | 62 | 4.0 | 486 | 62.6 | 4.4 | 0.111 |
| Harman et al, 2000 | | 72 | 64.8 | 8.9 | 127 | 65.7 | 9.7 | 0.518 |
| Kurek et al, 2000 | | 171 | 66.2 | 6.1 | 67 | 64.5 | 9.9 | 0.110 |
| Stattin et al, 2000 | | 149 | 59.7 | – | 298 | 59.6 | – | – |
| Khosravi et al, 2001 | | 84 | 64.8 | 6.2 | 75 | 65.6 | 6.0 | 0.411 |

– not available; *Student *t*-test; †Pearson chi-square test.

which controls consisted entirely of healthy males, and studies in which controls consisted entirely of patients with BPH, were 0.195, 0.194, 0.197, 0.153 and 0.296, respectively (Table 4). These HSMD were statistically significant ($P < 0.05$). The overall estimations of the HSMD for IGFBP-3 in the same groups were 0.111, 0.116, 0.100, 0.123 and 0.083 respectively, being statistically significant ($P < 0.05$) for the high-score group and studies in which controls consisted entirely of healthy males.

Table 4 also shows the estimated OR and its 95% CI with respect to IGF-I and IGFBP-3 in each study and in all studies combined, as well as analyses by the control type and study score. The OR for IGF-I was > 1.00 in 10 of the 14 studies. An OR significantly higher than 1.0 was found in 6 studies; 4 studies had an OR significantly less than 1.0 when comparing high levels IGF-I with low IGF-I. The overall OR for IGF-I was 1.47 (95% CI 1.27–1.71). There was no homogeneity among the 14 studies ($\chi^2 = 201.1$, degree of freedom (df) = 13, $P < 0.001$).

When 3 studies that used patients with benign prostatic hyperplasia as controls (Djavan et al, 1999; Finne et al, 2000, Khosravi et al, 2001) were excluded from the OR estimation, the overall OR declined from 1.47 to 1.36, but remained statistically significant ($P < 0.01$). The homogeneity test showed that the remaining 11 studies were still not homogeneous with regard to the effect size (chi-square = 71.03, df = 10, $P < 0.01$).

The overall ORs were 1.47 and 1.48 ($P < 0.01$ for both) for the high scoring and low-scoring groups respectively. Homogeneity

*© 2001 Cancer Research Campaign*

**Table 4** The individual and combined odds ratios (OR) and its 95% confidence interval (CI) by IGF-I and IGFBP-3

| | | | IGF-I | | | | | IGFBP-3 | | | | |
|---|---|---|---|---|---|---|---|---|---|---|---|---|
| **Citation** | **Score** | **Control** | **N1** | **N2** | **HSMD** | **OR** | **95% CI** | **N1** | **N2** | **HSMD** | **OR** | **95% CI** |
| Chan et al, 1998 | High | HM | 151 | 152 | 0.245 | 1.63 | 1.04, 2.54 | 152 | 152 | 0.007 | 1.01 | 0.65, 1.59 |
| Harman et al, 2000 | High | HM | 71 | 126 | 0.066 | 1.14 | 0.64, 2.03 | 71 | 126 | 0.000 | 1.00 | 0.56, 1.79 |
| Kurek et al, 2000 | High | HM | 171 | 67 | –0.008 | 0.98 | 0.56, 1.73 | 171 | 67 | – | – | – |
| Mantzoros, 1997 | High | HM | 51 | 52 | 0.556 | 3.00 | 1.38, 6.26 | 51 | 52 | – | – | – |
| Signorello et al, 1999 | High | HM | 208 | 70 | 0.124 | 1.28 | 0.75, 2.20 | 208 | 70 | 0.110 | 1.25 | 0.72, 2.13 |
| Stattin et al, 2000 | High | HM | 149 | 298 | 0.235 | 1.60 | 1.08, 2.36 | 149 | 298 | 0.203 | 1.50 | 1.01, 2.21 |
| Wolk et al, 1998 | High | HM | 210 | 224 | 0.217 | 1.54 | 1.06, 2.24 | 210 | 224 | 0.164 | 1.39 | 0.95, 2.02 |
| Baffa et al, 2000 | Low | HM | 57 | 39 | –0.513 | 0.36 | 0.17, 0.82 | 57 | 39 | – | – | – |
| Cohen et al, 1993 | Low | HM | 32 | 16 | 0.330 | 1.93 | 0.58, 6.09 | 32 | 16 | – 0.081 | 0.85 | 0.26, 2.80 |
| Ho and Baxter, 1997 | Low | HM | 12 | 7 | 0.282 | 1.75 | 0.28, 10.04 | 12 | 7 | 1.636 | 19.82 | 3.07, 84.69 |
| Kanety et al, 1993 | Low | HM | 14 | 6 | –2.644 | 0.01 | 0.00, 0.08 | 14 | 6 | – | – | – |
| Djavan et al, 1999 | Low | BPH | 71 | 174 | 1.668 | 20.82 | 12.64, 33.07 | 71 | 174 | – | – | – |
| Finne et al, 2000 | Low | BPH | 179 | 486 | –0.190 | 0.68 | 0.49, 0.96 | 179 | 486 | 0.025 | 1.05 | 0.75, 1.48 |
| Khosravi et al, 2001 | Low | BPH | 84 | 75 | 0.550 | 2.96 | 1.59, 5.39 | 84 | 75 | 0.274 | 1.73 | 0.92, 3.18 |
| Combined group | High score | | 1011 | 989 | 0.194 | 1.47 | 1.23, 1.77 | 790 | 870 | 0.116 | 1.26 | 1.03, 1.54 |
| | Low score | | 449 | 803 | 0.197 | 1.48 | 1.16, 1.90 | 307 | 584 | 0.100 | 1.22 | 0.91, 1.63 |
| | HM | | 1126 | 1057 | 0.153 | 1.36 | 1.14, 1.62 | 834 | 893 | 0123 | 1.28 | 1.05, 1.56 |
| | BPH | | 334 | 735 | 0.296 | 1.80 | 1.37, 2.36 | 263 | 561 | 0.083 | 1.18 | 0.87, 1.59 |
| | All studies | | 1460 | 1792 | 0.195 | 1.476 | 1.27, 1.71 | 1097 | 1454 | 0.111 | 1.248 | 1.06–1.47 |

N1: number of cases; N2: number of controls; HSMD: HSMD between cases and controls. The BPH patient and healthy male (HM) served as controls,
– Not available.

was found for the high-scoring group ($P = 0.355$) but not for the low-scoring group ($P < 0.001$). Since high scoring studies were homogeneous, it might be more reliable to use the overall OR from these studies to estimate the effect size. For the high-scoring group the odds of having prostate cancer was 1.47 (95% CI 1.23–1.77), when comparing men with high IGF-I with those with low IGF-I levels.

The overall OR for IGFBP-3 was 1.25 (95% CI 1.058–1.470) in the 9 studies, which were found to be homogeneous ($\chi^2 = 13.17$, df = 8, $P = 0.106$). Of the 9 studies, 2 studies used patients with BPH as controls (Finne et al, 2000; Khosravi et al, 2001) and when these were excluded, the overall OR increased from 1.25 to 1.28, remaining statistically significant ($P = 0.015$).

The overall estimation of OR for IGFBP-3 increased slightly from 1.25 to 1.26 for the high-scoring group (95% CI 1.03–1.54) and decreased from 1.25 to 1.22 for the low-scoring group (95% CI 0.91–1.63). The homogeneity test showed that the high score studies were homogeneous ($\chi^2 = 2.56$, df = 4, $P = 0.64$), whereas the low-scoring studies were not ($P = 0.012$).

### DISCUSSION

Selection bias may exist in case–control studies, and this bias, if it existed in the original study, cannot be controlled in meta-analysis. Out of the 7 studies, 5 (high-scoring group) included in the overall estimation of OR for IGF-I in the final analysis were matched (by age) case–control studies; this may lead to either a gain or loss in study efficiency and introduce selection bias. Matching is a useful tool for improving study efficiency in terms of the amount of information per subject studied, in some but not all situations (Rothman and Greenland, 1998). There was no homogeneity among the 14 studies included in the initial meta-analysis. Half of the studies would need to be excluded before we would be able to reach homogeneity. Even though there were only 7 studies left after exclusion, we still had a total of 2000 cases and controls in the final analysis. Overall, circulating IGF-I levels were higher in prostate cancer patients than in the control subjects, and the difference was statistically significant. An overall HSMD for IGF-I was 0.194, suggesting that the average level of IGF-I in the case group exceeds the levels of 57% of individuals in the control group. An overall OR for IGF-I was 1.47, suggesting that the odds of having prostate cancer are 1.47 times greater for men with high levels of IGF-I compared to those with low IGF-I levels.

Among the studies included in the meta-analysis, there were 3 laboratory methods used for IGF-I measurement and IGF-I concentrations varied substantially across the studies; in some cases, the difference was as high as 2-fold (Chan et al, 1998, vs Kurek et al, 2000). Despite this, it is unlikely that the significant difference in IGF-I between cases and controls was due to the different analytic methods for IGF-I, since standard mean differences were adjusted for variations in measurement methods.

Though sample size varied significantly between studies, this was adjusted in the analysis and would not affect the HSMD estimation.

Because age and other factors were matched between cases and controls in some of studies, the overall HSMD might not reflect very well the paired difference between case and control subjects in this meta-analysis. In our final analysis of IGF-I in high-scoring group, age as a confounding factor, was controlled for in the original study design. Our test showed that the age was not a statistical-significant difference in 4 out of the 7 studies (Table 3). The other 2 studies that did not report mean age for case and control (Chan et al, 1998; Stattin et al, 2000) were matched by age, therefore, a difference in IGF-I between the cases and controls might indeed be present.

In a case–control study, selection, recall and misclassification biases may exist. In addition, variation of study design was present in the 14 studies included in our meta-analysis. However, more

© 2001 Cancer Research Campaign than half of the studies were matched by age, smoking status, or residency, thereby minimizing bias.

The lack of control of other risk factors for prostate cancer could in theory have an effect on the results of our study. For example, the level of prostate-specific antigen (PSA) could be a confounder in this study. Men with elevated PSA levels were more likely to be subsequently diagnosed with prostate cancer than those with normal PSA levels (Gann et al, 1995; Chan et al, 1998). However, one study (Chan et al, 1998) showed that the plasma IGF-I level was strongly related to the risk of prostate cancer even among men with a normal baseline PSA level. Therefore, it is not likely that the PSA level has a confounding effect on the association between IGF-I and prostate cancer. Other confounding factors might exist, but in the framework of this study, they obviously could not be evaluated.

IGF-I is a peptide hormone with strong mitogenic effect on prostate cancer growth. In this meta-analysis, we found that IGF-I levels in the blood were higher in prostate cancer patients than in the controls. High circulating IGF-I may indicate an increase in prostate cancer risk. It would be interesting to know if serum IGF-I measurements have a role in the diagnosis of prostate cancer and a large prospective study would be worthwhile.

### ACKNOWLEDGEMENT

We thank Dr Mitch Harman and Dr June Chan for their invaluable assistance in obtaining the mean and standard deviation of IGF-I and IGFBP-3 in their studies.

### REFERENCES

Baffa R, Reiss K, El Gabry EA, Sedor J, Moy ML, Shupp-Byrne D, Strup SE, Hauck WW, Baserga R and Gomella LG (2000) Low serum insulin-like growth factor 1 (IGF-1): a significant association with prostate cancer. *Tech Urol* **6**: 236–239

Chan JM, Stampfer MJ, Giovannucci E, Gann PH, Ma J, Wilkinson P, Hennekens CH and Pollak M (1998) Plasma insulin-like growth factor-I and prostate cancer risk: a prospective study (see comments). *Science* **279**: 563–566

Cohen P, Peehl DM, Stamey TA, Wilson KF, Clemmons DR and Rosenfeld RG (1993) Elevated levels of insulin-like growth factor-binding protein-2 in the serum of prostate cancer patients. *J Clin Endocrinol Metab* **76**: 1031–1035

Cohen J (1965) Some statistical issues in psychological research. In: Wolman BB (ed.) *Handbook of Clinical Psychology,* McGraw-Hill, New York. pp 95–121

DerSimonian R and Laird N (1986) Meta-analysis in clinical trials. *Control Clin Trials* **7**: 177–188

Daniel W (1999) Biostatistics: A Foundation for Analysis in the Health Sciences, 7th edn, John Wiley & Sons, Inc. Series: New York, NY

Djavan B, Bursa B, Seitz C, Soeregi G, Remzi M, Basharkhah A, Wolfram R and Marberger M (1999) Insulin-like growth factor 1 (IGF-1), IGF-1 density, and IGF-1/PSA ratio for prostate cancer detection. *Urology* **54**: 603–606

Finne P, Auvinen A, Koistinen H, Zhang WM, Maattanen L, Rannikko S, Tammela T, Seppala M, Hakama M and Stenman UH (2000) Insulin-like growth factor 1 is not a useful marker of prostate cancer in men with elevated levels of prostate-specific antigen. *J Clin Endocrinol Metab* **85**: 2744–2747

Gann PH, Hennekens CH and Stampfer MJ (1995) A prospective evaluation of plasma prostate-specific antigen for detection of prostatic cancer. *JAMA* **273**: 289–294

Harman SM, Metter EJ, Blackman MR, Landis PK and Carter HB (2000) Serum levels of insulin-like growth factor I (IGF-I), IGF-II, IGF-binding protein-3, and prostate-specific antigen as predictors of clinical prostate cancer. *J Clin Endocrinol Metab* **85**: 4258–4265

Hedges LV and Olkin I (1985) Statistical methods for meta-analysis. Academic Press: San Diego, CA

Hedges LV (1994) Fixed effects models. In: Cooper H and Hedges LV (eds), *The Handbook of Research Synthesis* (pp 285–299). Rusell Sage Foundation: New York

Ho and Baxter (1997) Insulin-like growth factor-binding protein-2 in patients with prostate carcinoma and benign prostate hyperplasia. *Clin Endocrinol* **46**: 333–342

Jones JI and Clemmons DR (1995) Insulin-like growth factors and their binding proteins: biological actions. *Endocr Rev* **16** (1): 3–34

Juul A, Bang P, Hertel NT, Main K, Dalgaard P, Jorgensen K, Muller J, Hall K and Skakkebaek NE (1994) Serum insulin-like growth factor-I in 1030 healthy children, adolescents, and adults: relation to age, sex, stage of puberty, testicular size, and body mass index. *J Clin Endocrinol Metab* **78** (3): 744–752

Juul A, Dalgaard P, Blum WF, Bang P, Hall K, Michaelsen KF, Muller J and Skakkebaek NE (1995) Serum levels of insulin-like growth factor (IGF)-binding protein-3 (IGFBP-3) in healthy infants, children, and adolescents: the relation to IGF-I, IGF-II, IGFBP-1, IGFBP-2, age, sex, body, mass index, and pubertal maturation. *J Clin Endocrinol Metab* **80**: 2534–2542

Kanety H, Madjar Y, Dagan Y, Levi J, Papa MZ, Pariente C, Goldwasser B and Karasik A (1993) Serum insulin-like grwoth factor-binding protein-2 (IGFBP-2) is increased and IGFBP-3 is decreased in patients with prostate cancer: correlation with serum prostate-specific antigen. *J Clin Endocrinol Metab* **77**: 229–233

Khosravi J, Diamandi A, Mistry J and Scorilas A (2001) Insulin-like growth factor I (IGF-I) and IGF-binding protein-3 in benign prostatic hyperplasia and prostate cancer. *J Clin Endocrinol Metab* **86**: 694–699

Rothman K and Greenland S (1998) Modern epidemiology 2nd ed. Lippincott-Raven: Philadelphia, PA, USA

Kurek R, Tunn UW, Eckart O, Aumuller G, Wong J and Renneberg H (2000) The significance of serum levels of insulin-like growth factor-1 in patients with prostate cancer. *BJU Int* **85**: 125–129

Mantzoros CS, Tzonou A, Signorello LB, Stampfer M, Trichopoulos D and Adami HO (1997) Insulin-like growth factor 1 in relation to prostate cancer and benign prostatic hyperplasia. *Br J Cancer* **76**: 1115–1118

Prentice RL and Thomas DB (1986) on the epidemiology of oral contraceptives and disease. *Adv Cancer Res* **49**: 285–401

Rosenthal R and Rubin DB (1982) A simple general purpose display of magnitude of experimental effect. *Journal of Educational Psychology* **74**: 166–169

Rosenthal R (1994) Parametric measures of effect size. In: Cooper H and Hedges LV (eds), *Handbook of Research Synthesis* Russell Sage Foundation: New York

Signorello LB, Brismar K, Bergstrom R, Andersson SO, Wolk A, Trichopoulos D and Adami HO (1999) Insulin-like growth factor-binding protein-1 and prostate cancer. *J Natl Cancer Inst* **91**: 1965–1967

Stattin P, Bylund A, Rinaldi S, Biessy C, Dechaud H, Stenman UH, Egevad L, Riboli E, Hallmans G and Kaaks R (2000) Plasma insulin-like growth factor-I, insulin-like growth factor-binding proteins, and prostate cancer risk: a prospective study. *J Natl Cancer Inst* **92**: 1910–1917

Wolk A, Mantzoros CS, Andersson SO, Bergstrom R, Signorello LB, Lagiou P, Adami HO and Trichopoulos D (1998) Insulin-like growth factor 1 and prostate cancer risk: a population-based, case-control study. *J Natl Cancer Inst* **90**: 911–915

© 2001 Cancer Research Campaign
